# Whole Genome Analysis Reveals Evolutionary History and Introgression Events in Bale Monkeys

**DOI:** 10.3390/genes15111359

**Published:** 2024-10-23

**Authors:** Lakshmi Seshadri, Anagaw Atickem, Dietmar Zinner, Christian Roos, Liye Zhang

**Affiliations:** 1 Primate Genetics Laboratory, German Primate Center, Leibniz Institute for Primate Research, 37077 Göttingen, Germany; lseshadri@dpz.eu; 2 International Max Planck Research School for Genome Science (IMPRS-GS), Georg-August-Universität Göttingen, 37077 Göttingen, Germany; 3 Department of Zoological Sciences, Addis Ababa University, Addis Ababa 999047, Ethiopia; anagawam@gmail.com; 4 Cognitive Ethology Laboratory, German Primate Center, Leibniz Institute for Primate Research, 37077 Göttingen, Germany; dzinner@gwdg.de; 5 Department of Primate Cognition, Georg-August-Universität Göttingen, 37077 Göttingen, Germany; 6 Leibniz ScienceCampus Primate Cognition, 37077 Göttingen, Germany; 7 Gene Bank of Primates, German Primate Center, Leibniz Institute for Primate Research, 37077 Göttingen, Germany

**Keywords:** *Chlorocebus djamdjamensis*, African green monkey, gene flow, adaptation, high altitude, diet specialization

## Abstract

**Background/Objective:** The Bale monkey (*Chlorocebus djamdjamensis*) is a threatened primate species endemic to Ethiopia and, in contrast to other members of the genus *Chlorocebus*, lives at high altitudes and feeds mainly on bamboo. Two populations of the species are present, one in continuous bamboo forest (CF) in the eastern part of the species’ range, and the other in fragmented forest (FF) in the western part. Based on mitochondrial DNA and phenotypic characteristics, previous studies have suggested introgression by parapatric congeners into the FF population but not into the CF population. The objective of this study was to gain insights into the evolutionary history of Bale monkeys and their potential genetic adaptations to high altitudes and for bamboo consumption. **Methods:** We sequenced the whole genomes of individuals from both populations and compared their genomes with those of the other five *Chlorocebus* species. We applied phylogenetic methods and conducted population demographic simulations to elucidate their evolutionary history. A genome-wide analysis was conducted to assess gene flow and identify mutations potentially associated with adaptations to high altitudes and for bamboo metabolism. **Results:** Our analyses revealed Bale monkeys as the sister clade to *Chlorocebus aethiops* and showed that gene flow occurred between *C. aethiops* and FF but not between *C. aethiops* and CF. In addition, we detected non-synonymous mutations in genes potentially associated with the adaptation to high altitudes (*EPAS1*) in both populations and with the adaptation for bamboo metabolism (*TAS2R16*, *MPST*, and *TST*) mainly in the CF population. **Conclusions:** Our study provides insights into the evolutionary history of a threatened primate species and reveals the genetic basis for its adaptions to unique environments and for diet specialization.

## 1. Introduction

Hybridization, the gene flow between genetically distinct populations producing offspring of mixed ancestry [1], is one of the most interesting topics in evolutionary biology [2,3,4]. Recent research highlights hybridization as a powerful evolutionary driving force, affecting species diversity and phenotypic traits across plants and animals [5,6,7,8,9].

The effects of hybridization can vary, ranging from detrimental outcomes such as a loss of diversity to beneficial effects like introducing genetic diversity, enhancing adaptation, or hybrid speciation [4,8,10,11]. Hybridization has affected the taxa of all major primate radiations, including humans, and African green or savannah monkeys (the genus *Chlorocebus*) are no exception [12,13,14,15,16,17,18]. The genus *Chlorocebus* comprises six species, widely distributed in the savannah–woodlands and riverine forests of sub-Saharan Africa [19]. They are omnivores and more terrestrial than other guenons [20]. Incongruences between mitochondrial, Y-chromosome, and phenotype-based phylogenies suggest gene flow among members of the genus [21,22,23]. Genomic studies that included five of the six species confirmed ancient gene flow between various *Chlorocebus* lineages [15,18]. *C. djamdjamensis* was not included in these studies, but mitochondrial paraphylies suggest that interspecific gene flow has also affected this member of the genus [21,23].

**Figure 1:**
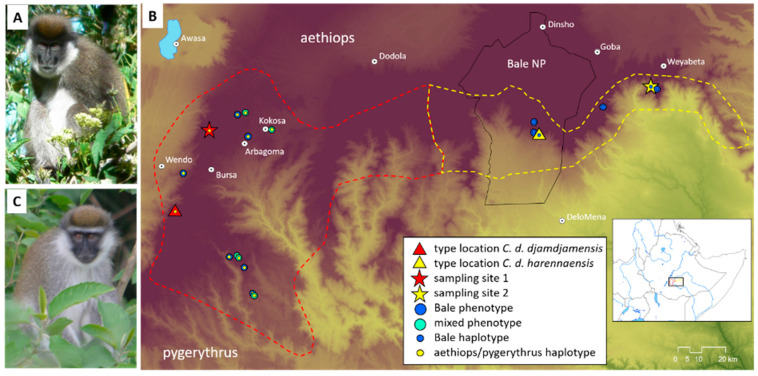
Photographs and distribution map including sampling sites of Bale monkeys. (**A**) *C. djamdjamensis* from Odobullu Forest (continuous forest, CF). (**B**) Distribution range of *C. djamdjamensis* and geographic origin of samples. Red dashed line = approximate range of *C. d. djamdjamensis* (fragmented forest, FF); yellow dashed line = approximate range of *C. d. harennaensis* (continuous forest, CF). Triangles = type localities of the two subspecies; stars = geographic origin of sequenced individuals; large blue circles = geographic origin of individuals with typical *C. djamdjamensis* phenotype; large turquoise circles = geographic origin of individuals with intermediate phenotype; small blue circles = *C. djamdjamensis* mitochondrial haplotype; yellow small circles = *C. aethiops*/*pygerythrus* mitochondrial haplotypes. The inserted map shows the position of the study area within Ethiopia (the map was produced using ArcMap (version 10); species distribution outline comes from IUCN). (**C**) *C. djamdjamensis* with intermediate phenotype from Sidamo Highlands (fragmented forest, FF) (note white hair stripe above the eyebrows).

The Bale monkey (*C. djamdjamensis*; Figure 1A), unlike its five widely distributed congeners, occupies a restricted range in the Ethiopian Highlands southeast of the Rift Valley (Figure 1B) [20,24]. While other *Chlorocebus* species live up to altitudes of 1800 m above sea level (asl) and are predominantly frugivorous [20], *C. djamdjamensis* inhabits higher altitudes (up to 3400 asl) [24] and feeds largely on bamboo [25]. Previous studies have shown that species living at high altitudes have accumulated mutations in genes related to lung function, DNA repair, and angiogenesis, which alter protein function and allow them to persist in high-altitude environments [26,27,28,29,30,31,32,33,34]. Likewise, species that feed on bamboo have evolved mechanisms to perceive and detoxify harmful cyanides that can occur in high concentrations in bamboo [35].

Due to differences in pelage coloration and their membership in different mitochondrial haplogroups, Gippoliti [36] and Butynski et al. [37] recognized two subspecies, with *C. djamdjamensis djamdjamensis* occurring in the Sidamo Highlands, i.e., the western part of the species range, and *C. djamdjamensis harennaensis* in the Bale Mountains, the eastern part. Human activities such as the expansion of agriculture and settlements have led to a fragmentation of forests in the western part of the species’ range, whereas the habitat in the eastern part is mainly characterized by continuous montane bamboo forest [38,39]. The species is listed as Vulnerable by the IUCN [37], and both subspecies are listed as Endangered [40,41]. Besides individuals with the typical *C. djamdjamensis* phenotype (Figure 1A), individuals showing phenotypic traits of parapatric grivet (*C. aethiops*) or vervet monkeys (*Chlorocebus pygerythrus*) (Figure 1C) also occur in the western part of their range [38]. This, in combination with the geographic distribution pattern of mitochondrial haplotypes, suggests that introgression occurred between *C. djamdjamensis* and grivet or vervet monkeys [23,42].

There is evidence that the two *C. djamdjamensis* populations, the eastern continuous forest population (CF) and the western fragmented forest population (FF), are differentially affected by hybridization, but no genomic data are available to investigate this hypothesis. Therefore, we generated whole genome data from one individual of each population and compared them with the published genome data of the other five *Chlorocebus* species [15]. In our study, we aimed to settle the phylogenetic position of *C. djamdjamensis* among African green monkeys, model their demographic history, find traces of introgression from parapatric *C. aethiops* or *C. pygerythrus*, and identify potential genomic adaptations of *C. djamdjamensis* to high altitudes and for diet specialization, i.e., bamboo consumption.

## 2. Materials and Methods

### 2.1. Sample Collection and Sequencing

Two *C. djamdjamensis* individuals, one from Odobullu Forest of the Bale Mountains and one from Bensa Forest in the Sidamo Highlands, were trapped with box traps baited with bananas and maize. No monkey was injured during the trapping and handling procedure. Animals were anaesthetized by experienced veterinarians using a combination of 0.05 mg/kg dexmedetomidine followed by 5 mg/kg ketamine, while a combination of 5 mg/kg of atipamezole per 0.5 mg of dexmedetomidine was used for reversal. Whole blood (5 mL) was drawn from the Vena femoralis, stored in 5 mL EDTA-K3 tubes and sent to the genetics lab of the German Primate Center. We extracted DNA with the First-DNA all-tissue Kit (GEN-IAL, Troisdorf, Germany) following the manufacturer’s instructions and measured DNA concentrations with Qubit 3.0 (ThermoFisher Scientific, Waltham, MA, USA). We sent 200 ng of DNA to Eurofins (Ebersberg, Germany) for whole genome sequencing using HiSeq 4000 (150 bp pair-end reads; Illumina, San Diego, CA, USA).

### 2.2. Mitochondrial Genome Assembly and Phylogeny

We extracted complete mitochondrial genomes (mitogenomes) of the two *C. djamdjamensis* individuals from the raw sequence data using the Geneious Prime 2023.0.1 package https://www.geneious.com/ (accessed on 13 April 2023). We first trimmed and quality-filtered the reads with BBDuk (version 38.84) of the BBTools package implemented in Geneious and then removed duplicate reads with Dedupe (version 38.84) (BBTools package), both with default settings. Clean reads were then mapped onto the putatively closest available mitogenome (NC_034277 for CF and NC_034276 for FF) using the Geneious Assembler with default settings (maximum of 5 iterations). Generated mitogenomes were manually checked and annotated in Geneious.

For phylogenetic tree reconstruction, we expanded our dataset with ten additional *Chlorocebus* mitogenomes and added *Cercopithecus mitis* as an outgroup. The 13 mitogenomes were aligned with Muscle (version 3.8.31) [43] in AliView (version 1.18) [44]. We generated a maximum likelihood (ML) tree, treating the mitogenome as a single partition, with IQ-TREE (version 2.2.0) [45] using 10,000 ultrafast bootstrap replications [46] and the best-fit substitution model (TIM2 + F + I + G4) as determined by ModelFinder [47] according to the Bayesian information criterion (BIC). The generated tree was visualized with FigTree (version 1.4.4) http://tree.bio.ed.ac.uk/software/figtree/ (accessed on 20 April 2023).

### 2.3. Additional Whole Genome Data

We expanded our dataset with whole genome sequence data of representatives of the other five *Chlorocebus* species (four individuals of *Chlorocebus sabaeus*, six individuals each of *C. aethiops*, *Chlorocebus cynosuros*, and *Chlorocebus tantalus*, four individuals of *C. pygerythrus hilgerti*, and 14 individuals of *C. pygerythrus pygerythrus*) previously published by Svardal et al. [15] (Table S1). These samples were chosen based on their geographical location and sequencing depth of at least 3×.

### 2.4. Mapping and Variant Calling

In the initial data-processing phase, we performed quality control using FastQC (version 0.12) [48]. Raw sequence reads were then adapter-trimmed and quality-filtered with fastp (version 0.23.4) [49] with the following criteria: reads with unidentified nucleotides (N) exceeding 10% and reads with a proportion of low-quality bases (Phred quality ≤ 5) greater than 50% were discarded. After quality control, 40.1 gigabase pairs (Gbp) and 37.1 Gbp of high-quality sequences with 269.9 and 252.0 million pair-end reads (150 bp) were generated for FF and CF, respectively. High-quality reads were mapped to the *Macaca mulatta* (Mmul_10) and *C. sabaeus* (chlSab2) reference genomes using the Burrow–Wheeler alignment implemented using BWA-MEM algorithm (version 0.7) [50] (Table S1). Notably, secondary reads of shorter lengths were marked, and supplementary alignments were denoted as soft clipping [51]. The sequence alignment mapping file (.sam) was converted to binary files (.bam) with SAMtools (version 1.12) [52]. The mapped files were then sorted based on coordinate numbers, and duplicate reads arising from PCR amplification were marked using Picard (version 3.0) (http://broadinstitute.github.io/picard, accessed on 13 April 2023). Additionally, we built indexes for BAM files using SAMtools. Post-alignment quality control procedures were performed with Mosdepth (version 0.34) [53] to assess the coverage and depth across the mapped genome.

The haplotype calling process was conducted using the Genome Analysis Toolkit (GATK version 4.2) following best practices. A sequence dictionary and FASTA index file were generated for the *M. mulatta* reference genome. Variants were called using GATK HaplotypeCaller, and subsequent filtering was performed through the VariantFilteration functionality of GATK. Filters included QD < 2, FS > 60, MQ < 40, HaplotypeScore > 13, MQRankSum < −12.50, and ReadPosRankSum < −8.00, specifically for single-nucleotide polymorphisms (SNPs). To validate the called variants, BCFtools (version 1.18) [54] was employed, and only SNPs were retained in the final merged VCF file for subsequent analysis. We measured heterozygosity in autosomes with PLINK (version 2.0.0) [55] using SNP data and the ‘--het’ parameter.

### 2.5. Principal Component Analysis

We conducted a principal component analysis (PCA) to investigate the relationships among *C. djamdjamensis* from CF and FF with 40 other *Chlorocebus* samples. Merged and filtered VCF files underwent pre-processing using VCFtools (version 0.19) [56] with the following parameters: --minDP = 4 and --minQ = 40. The VCF index was built using BCFtools. The pre-processed VCF files were transformed into a PLINK-compatible format using ‘--plink’ option in VCFtools. We performed PCA using PLINK (version 1.9) with ‘--make-bed’ to create binary PED files and ‘--pca’ to derive eigenvalues and eigenvectors. The significance of eigenvectors was determined with the Tracy–Widom test in R (version 4.3.1) (AssocTests package) [57,58].

### 2.6. Autosomal Phylogeny

To generate the whole genome FASTA and PHYLIP sequence of all samples, we ran a Perl script to transform the merged VCF file. A phylogenetic tree was reconstructed with the ML algorithm using IQ-TREE (version 2.2.0) [45]. The best-fit nucleotide substitution model (TVM + F + G4) was selected using BIC as implemented in IQ-TREE, and 10,000 ultrafast bootstrap replications were performed to obtain nodal support. Trees were visualized with FigTree (version 1.4.4) (http://tree.bio.ed.ac.uk/software/figtree/, accessed on 20 April 2023).

### 2.7. Demographic History

Changes in effective population size for each taxon were inferred from the haploid pairwise sequential Markovian coalescence (PSMC) model [59], which utilizes the patterns of heterozygosity along a single genome and a hidden Markov model to reconstruct the fluctuations of effective population size over time. Given the sensitivity of PSMC analysis to the total number of genomic positions [60], we specifically selected medium-coverage *Chlorocebus* individuals (3–6× sequencing depth). The analysis was performed with autosomal data.

Our *C. djamdjamensis* genome data exhibited a higher level of coverage than other *Chlorocebus* individuals; hence, we down-sampled them using Picard to 4× depth to standardize sequencing depth across all *Chlorocebus* samples. Diploid sequences were generated through SAMtools (version 1.8), BCFtools (version 1.10.2), and vcfutils.pl script using the BAM files mapped to *M. mulatta* that we obtained by ANGSD (version 0.933) (low-coverage mapping) [61]. For each individual, we followed the recommended pipeline in the PSMC manual to perform SAMtools mpileup and BCFtools view analysis to generate the diploid FASTQ sequence. The diploid consensus genome sequence was generated using vcfutils.pl vcf2fq program (a part of the BCFtools distribution) with the following parameters: -d 4 -D 300. The consensus sequence was transformed by fq2psmcfa with -q 20. PSMC analysis was set as follows: “4 + 25 × 2 + 4 + 6”, 25, 15, 5, and 20, respectively. A generation time (-g) of 8.5 and a mutation rate (-u) of 1.5 × 10^−8^ per site per generation were adopted from Warren et al. [62] and Svardal et al. [15]. For bootstrapping, we ran the PSMC with 100 replications for each sample to simulate a realistic population history and assess the variation in the inferred *N_e_* trajectories.

### 2.8. Gene Flow Analysis

To investigate if one or both *C. djamdjamensis* individuals are affected by introgression from other parapatric *Chlorocebus* species, we performed *D*-statistics using Dtrios of Dsuite [63] based on all autosomal alleles. In our analysis, we set *C. sabaeus* as the outgroup O. *D*-statistics is calculated by systematically evaluating triplet combinations within groups of populations/samples. In Dsuite, a total of 36 combinations were tested and filtered (*p* value < 0.05; −3 > Z-score > 3), and gene flow percentages were calculated according to Svardal et al. [15]. Next, we performed *f*_4_ analysis [63] based on *f*_4_ ratio [13] with Dsuite, which evaluates allele frequency covariances among four samples (P1, P2, P3, and O as the outgroup) taking into account the phylogenetic relationship among taxa. We utilized autosomal SNP data to estimate *f*_4_ statistics, as it is robust under most demographic scenarios and can identify traces of excess allele sharing. Additionally, to identify gene flow at the chromosome level, we performed a three-taxon *D_FOIL_* [64] approach for CF, FF, and *C. aethiops* with *C. sabaeus* as the outgroup.

DensiTree (version 3.0) [65] was applied to elucidate different branching patterns. We initially partitioned the entire genome into sliding windows using SeqKit (version 2.3) [66] with a window size of 500 kb with 250 kb steps and 50 kb with 25 kb steps [67]. We then generated ML trees for each window with IQ-TREE using the following parameters: --date-tip 0, --date-ci 100, and the alignment file from VCF using custom scripts. We merged the nexus output for window-based trees, and a custom Perl script was implemented to infer branching patterns of *C. aethiops* with CF and FF. We set the window sizes and steps to balance the resolution of the analysis while considering computational efficacy [68]. Furthermore, a window-based approach employing 500 kb windows was conducted with an ABBA-BABA python script following the methodology outlined by Martin et al. [69]. First, we ran parseVCF.py to exclude indels from the analysis by the parameter “skipIndels”. Then we applied the ABBA-BABA python script from the package, configuring a window size (-w) of 500 kb and a step size (-s) of 250 kb. This window-based approach facilitated the identification of overlapping regions with significant *D*-statistics, with emphasis on the top 5% overlapping windows. We used the Quantifying Introgression via Branch Lengths (QuIBL) method [70] to identify if both introgression and incomplete lineage sorting (ILS) or only ILS are responsible for these patterns. Gene trees served as input (see DensiTree methods). A total of 50 expectation–maximization (EM) steps were executed.

### 2.9. Gene Enrichment and Adaptation Analysis

SNP variant files for individual samples were annotated using snpEff (version 5.2) [71] based on the *C. sabaeus* (chlSab2) database provided by snpEff. Non-synonymous mutations were extracted from the VCF files for both *C. djamdjamensis* using the top outliers identified by ABBA-BABA window-based methods (above). Enrichment analysis was performed using KOBAS-i (KEGG Orthology-Based Annotation System) (Fischer’s exact test; Bonferroni correction; corrected *p* < 0.05) [72] to provide functional annotation and interpret the biological context of genes with non-synonymous mutations. *C. djamdjamensis* genomes were visualized with Integrative Genomics Viewer (IGV) (version 2.17.4) [73]. Furthermore, we performed literature searches to identify candidate genes potentially related to high altitude and bamboo metabolism in vertebrates [28,29,33,34,74,75,76,77,78,79,80,81]. According to the candidate gene list, we checked all non-synonymous mutations in these genes and aligned the sequences using ClustalW (version 2.1) [82] in MEGA (version 11) [83].

## 3. Results

### 3.1. Sampling and Sequencing

We generated whole genome data from two phenotypically pure *C. djamdjamensis* individuals with an average sequencing depth of 10.5× (CF) and 11.8× (FF) and with a genome coverage of 93.0% (mapped to the *M. mulatta* reference genome (Mmul_10)) for both individuals (Table S1). The merged VCF file consisting of 42 samples, the two *C. djamdjamensis* individuals, and 40 additional representatives of the genus *Chlorocebus* (Table S1) contained 67,048,876 high-quality SNPs, which were used for subsequent analyses. The autosomal heterozygosity values, measured as the number of heterozygous sites per 1000 bp, were 0.68 and 1.11 for the CF and FF individuals, respectively. The mean values for the other *Chlorocebus* species ranged between 0.76 and 0.87 (Table S2).

### 3.2. Mitochondrial Genome Assembly and Phylogeny

We generated complete mitogenomes of both *C. djamdjamensis* individuals with a high average sequencing depth (1024× for CF and 219× for FF) and lengths of 16,429 bp and 16,385 bp for CF and FF, respectively. The reconstructed ML tree, which included ten published mitogenomes of congeners, was well resolved, with most nodes gaining strong support values (bootstrap values: 83–100%) (Figure S1). The tree topology was identical to that presented by Dolotovskaya et al. [42] and depicted most *Chlorocebus* species as para- or polyphyletic clades. As expected by their geographic origin, CF clustered with clade C5 and FF with clade C2 of Haus et al. [21] and Dolotovskaya et al. [42]. The mitogenomes of CF and FF exhibit sequence similarity values to their respective reference mitogenomes, NC_034277 and NC_034276, of 99.31% and 99.79%, respectively.

### 3.3. Phylogeny and Demographic History Based on Whole Genome Data

**Figure 2:**
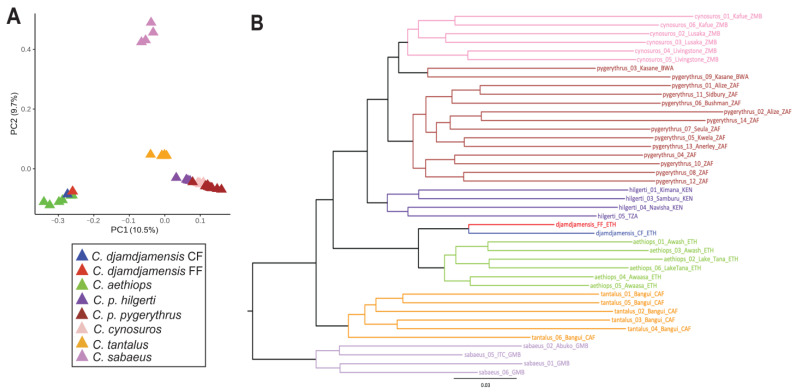
Principal component analysis (PCA) and phylogeny. (**A**) The PCA shows the first two components, with 10.5% and 9.7% of the variance explained by principal components 1 and 2, respectively. (**B**) Maximum likelihood (ML) tree based on autosomal single-nucleotide polymorphisms (SNPs), with tips indicating species names, sample ID, location of sample collection, and country of origin (BWA = Botswana, CAF = Central African Republic, ETH = Ethiopia, GMB = Gambia, KEN = Kenya, ZAF = South Africa, and ZMB = Zambia). The scale bar represents substitutions per site.

The PCA revealed four main clusters: (1) *C. sabaeus*, (2) *C. tantalus*, (3) *C. aethiops* + *C. djamdjamensis*, and (4) *C. pygerythrus* + *C. cynosuros* (Figure 2A and Figure S2). Principal component (PC) 1 explained 10.5% of the variance (Tracy–Widom; *p* < 0.05) and separated the cluster containing *C. aethiops* and *C. djamdjamensis* from all the others, while PC2 (which explained 9.7% of the variance) separated *C. sabaeus* and also, to some degree, *C. tantalus* from the others. The *C. aethiops* + *C. djamdjamensis* cluster and the *C. p. pygerythrus* + *C. cynosuros* cluster showed only little substructure and no clear differentiation into individual species.

We reconstructed an ML tree (Figure 2B) using autosomal SNP data and obtained robust nodal support (99–100%) across all branches. Species appeared as monophyletic clades, except for two *C. p. pygerythrus* individuals that clustered with *C. cynosuros*. *C. sabaeus* diverged first, followed by *C. tantalus* and the clade with *C. aethiops* and *C. djamdjamensis*. Among the remaining taxa, *C*. *p. hilgerti* formed a sister clade to *C. p. pygerythrus* and *C. cynosuros*.

**Figure 3:**
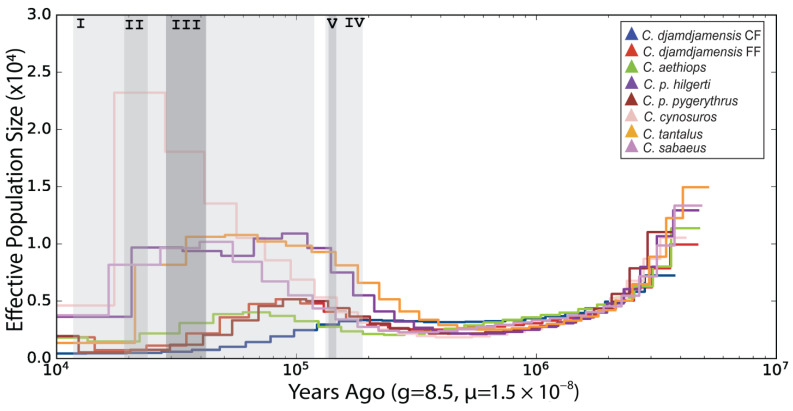
PSMC plots for *Chlorocebus* species, showing effective average population changes, including the CF and FF lineages. Grey shaded areas represent (I) last glacial period, (II) last glacial maximum, (III) last glacial maximum in the Bale Mountains, (IV) penultimate glacial period, and (V) penultimate glacial maximum. For individual PSMC plots, see Figure S3.

The results of the PSMC analysis suggest a shared population history of all six *Chlorocebus* species until 500 thousand years ago (kya) (Figure 3; for individual species plots, see Figure S3). Between 400 and 300 kya, the demographic histories of the species became more independent with varying increases in their effective population sizes. The species have most likely been differentially affected by the last glacial cycles. CF started to decrease during the penultimate glaciation (192–135 kya; IV in Figure 3), reached its low stand during the last maximum glaciation of the Bale Mountains (42–28 kya; III in Figure 3) [84] and remained at this low level. The other *Chlorocebus* lineages, including FF, continued to increase their effective population sizes until the last glacial period (115–11.7 kya; I in Figure 3). During or shortly after the last glacial maximum (LGM, 24.5–18 kya; II in Figure 3), all of the species reached a demographic low stand. In contrast to CF, FF slightly increased again after the LGM.

### 3.4. Unveiling Gene Flow Events

**Figure 4:**
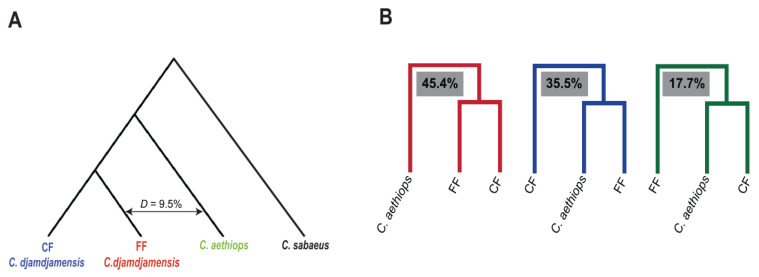
Gene flow analysis. (**A**) Whole genome D-statistics illustrating gene flow percentage between FF and *C. aethiops*. (**B**) Frequency of observed branching patterns between *C. aethiops*, CF, and FF based on 500 kb windows.

To test for gene flow, we used statistical and window-based approaches. Whole genome *D*-statistics indicated gene flow between *C. aethiops* and FF (9.5%) but not between *C. aethiops* and CF (Figure 4A; Table S3). Additionally, we also found evidence for gene flow among other *Chlorocebus* taxa (Table S3). These results were generally corroborated by positive *f*_4_ values as revealed by the *f*_4_ ratios from the Dsuite analysis [63] (Table S3). Specifically, we found a significant *f_4_* value for the combination of *C. aethiops* and FF but not for *C. aethiops* and CF, indicating that a substantial portion of the FF genome is affected by gene flow from *C. aethiops* (Table S3). The *D*-statistics and *f_4_* value are supported by a Z-score of 15.33 and a *p*-value of 2.30 × 10^−16^ for the gene flow between *C. aethiops* and FF, indicating that the likelihood for the occurrence of random gene flow is very low. This provides strong statistical evidence for gene flow between *C. aethiops* and FF. Furthermore, to test for gene flow at the chromosome level, we performed a *D_FOIL_* analysis for the trio ‘CF-FF-*C. aethiops*’ and found consistent evidence for gene flow between FF and *C. aethiops* across all chromosomes (Table S4).

In our window-based approach, we reconstructed ML trees for overlapping windows that were 500 kb and 50 kb in size and visualized them in DensiTree (Figure S4) [65]. When using 500 kb windows, in 45.4% of the trees, CF and FF formed a clade with *C. aethiops* as the sister taxon, while in 35.5% of the trees, *C. aethiops* clustered with FF, and CF was a sister to them. In 17.7% of the trees, *C. aethiops* grouped with CF, and FF was the sister taxon (Figure 4B). For 50 kb windows, we obtained similar results (Figure S5).

The QuIBL analysis [70] indicated that a combination of introgression and ILS is more likely to explain the branching of *C. aethiops* with FF than using only ILS (Table S5). Particularly *C. aethiops* individuals ‘aethiops_04’ and ‘aethiops_05’, which are geographically the closest to FF, showed the highest values (Table S5).

### 3.5. Annotation, Gene Enrichment, and Adaptation

**Figure 5:**
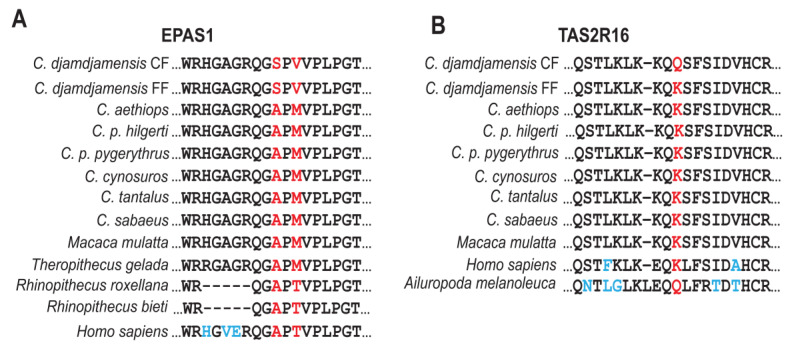
Amino acid changes in genes related to high altitude and bamboo consumption. (**A**) Partial amino acid alignment of the endothelial PAS domain protein 1 (EPAS1), associated with high-altitude adaptation, with unique amino acid changes (A665S and T/M667V) in CF and FF. (**B**) Partial amino acid alignment of the taste 2 receptor member 16 (TAS2R16), associated with bamboo consumption, showing a unique amino acid change (K68Q) in CF. Non-synonymous mutations present in CF and/or FF are marked in red, while mutations found in other species are marked in blue.

We made variant annotations and noticed clear differences in the number of variants across different mutation effect categories between CF and FF (Table S6). We further investigated non-synonymous mutations unique to *C. djamdjamensis* and compiled a curated list of genes previously associated with adaptations to high altitudes and for bamboo consumption in other species (Table S7). Based on an enrichment analysis of genes in regions with significant *D*-statistic values (the top 5%, corrected at a level of *p* < 0.05, for CF and FF) in KOBAS (KEGG Orthology-Based Annotation System) [85], we found six genes (*EGLN1*, *VEGFB*, *ITPR2*, and *MYB*) that were significantly enriched for the Gene Ontology (GO) terms ‘Response to Hypoxia’ and the KEGG (Kyoto Encyclopedia of Genes and Genomes) pathway ‘HIF-1 Signalling’ (Table S8). Similarly, we found genes enriched for the GO terms ‘Angiogenesis’ (*VEGFB, SOX17*, and *ANGPTL1*) and ‘Response to Oxidative Stress’ (*LRRK2, MBL2,* and *DUOX2*) (Tables S9 and S10). Our analysis revealed novel shared mutations (Ala665Ser and Thr/Met667Val) in the *EPAS1* gene (endothelial PAS domain protein 1) in both CF and FF (Figure 5A). Furthermore, we identified a mutation (Lys68Gln) in the *TAS2R16* (taste receptor 2 member 16) gene associated with the ‘Taste Transduction Pathway’ (hsa04742 and K08474) specifically in CF (Figure 5B). Additional non-synonymous mutations in genes related to cyanide detoxification, including *TST* and *MPST*, occurred mostly in CF (Figures S6 and S7).

## 4. Discussion

### 4.1. Phylogeny and Demographic History

The PCA, capturing the genetic variation among the investigated *Chlorocebus* individuals, is largely in agreement with the results of previous studies [15,18,62,86]. Our PCA results suggest four clusters: (1) *C. sabaeus*, (2) *C. tantalus*, (3) *C. aethiops* + *C. djamdjamensis*, and (4) *C. cynosuros* + *C. pygerythrus*. This clustering reflects the genetic similarity among *Chlorocebus* species, but it does not match with the six-species taxonomy of *Chlorocebus* [19,20]. Our ML phylogeny gives a more fine-grained picture and reveals seven well-supported clades that largely refer to species. The two west and central African clades, *C. sabaeus* and *C. tantalus*, diverged first, followed by the northeast African *C. aethiops* clade and its sister clade *C. djamdjamensis* (CF and FF) and the eastern and southern African taxa *C. p. hilgerti*, *C. p. pygerythrus,* and *C. cynosuros*. As a deviation from the six-species taxonomy, *C. p. hilgerti* forms a well-supported clade distinct from *C. p. pygerythrus* which may justify upgrading this taxon to species level. On the other hand, we note that the two *C. p. pygerythrus* individuals (pygerythrus_03 and pygerythrus_09) from Botswana, whose range is geographically close to the range of *C. cynosuros* from Zambia, clustered with *C. cynosurus* and not with *C. p. pygerythrus*. This geographic proximity raises the possibility that gene flow occurred or that the two specimens were phenotypically misidentified, warranting further investigation into their genetic and phenotypic characteristics and a detailed study of the range boundaries between the two species.

Our PSMC analysis reveals differential dynamics in the effective population sizes of the *Chlorocebus* taxa, suggesting that global climate changes, such as glacial periods, had an impact on all *Chlorocebus* taxa. In particular, the CF population was most likely strongly affected by the glaciation of their high-altitude habitat when large parts of the Bale and Arussi Mountains were covered by glaciers [84]. The size of the CF population began to decline during the penultimate glacial maximum (140 kya), reached its low stand during the LGM in the Bale Mountains (42–28 kya) [84], and has not recovered since then. Our data therefore do not support the hypothesis that the decline in the *C. djamdjamensis* populations began with the start of agriculture and other anthropogenic activities in their range [38,39]. In contrast, the effective population size of FF dropped significantly at the beginning of the last glacial period and reached its lowest point during the LGM. It recovered slightly at the beginning of the Holocene. However, this trajectory may also have been affected by gene flow from invading *C. aethiops,* resulting in an increase in genetic diversity and thus in effective population size.

### 4.2. Gene Flow Among Parapatric Species

Our analysis revealed gene flow between pairs of parapatric *Chlorocebus* taxa, which is largely in agreement with findings from previous studies [15,18,62]. These studies have identified ancient gene flow between *C. p. hilgerti* and *C. tantalus,* between *C. p. hilgerti* and *C. p. pygerythrus,* and between *C. p. pygerythrus* and *C. cynosuros* [15,18]. Furthermore, *C. cynosuros* was identified as a mixture between *C. p. hilgerti* and *C. p. pygerythrus*, and *C. p. pygerythrus* individuals from Botswana, which are sister to *C. cynosuros*, are intermediates between *C. cynosuros* and *C. p. pygerythrus* [15,62]. In general, the pattern of a complex demographic history with notable instances of gene flow found in *Chlorocebus* largely resembles that of other savannah species, such as baboons or geladas in Ethiopia [7,33,87,88].

Previous mitochondrial and phenotypic data have suggested both historical and contemporary gene flow between *C. aethiops* and FF but not between *C. aethiops* and CF [21,39]. Our whole genome analyses support this hypothesis. First, we find higher heterozygosity in the FF individual compared to the CF individual. In general, higher heterozygosity can be the result of gene flow, which increases genetic diversity by combining previously separated and diverged gene pools [89]. Second, window-based approaches show that FF shares more ancestry with *C. aethiops* than CF does. Third, the results from the whole genome and chromosome-based *D*-statistics are in line with these findings and indicate gene flow between *C. aethiops* and FF but not between *C. aethiops* and CF. Fourth, the *f*_4_ ratio provides strong evidence for gene flow between FF and *C. aethiops* (Table S3). Fifth, the QuIBL analysis confirms that the observed patterns are not solely due to ILS but also involve introgression. Particularly, *C. aethiops* individuals from Awasa (aethiops_04 and aethiops_05), whose range is geographically closest to the range of FF (Table S1), exhibit the strongest signals for gene flow with FF. Overall, the results of our analyses and previous studies of the mitochondrial relationships on a larger number of individuals of both populations indicate gene flow between *C. aethiops* and FF, while gene flow appears to be limited between CF and *C. aethiops*. Nevertheless, these findings should be interpreted with caution, as the observed excess allele sharing could be influenced by factors such as ancient population structure, sample size, selection, and genetic drift. However, given that individuals with intermediate phenotypes between Bale and grivet monkeys have already been reported in the FF population and considering the high human pressure to open up the continuous forest of the CF population, a possible scenario is that also the CF population will be introgressed and genetically swamped, and eventually the Bale monkey geno- and phenotypes will go extinct [23].

### 4.3. Potential Ecological Adaptations

Our enrichment analysis revealed in *C. djamdjamensis* non-synonymous mutations in genes known to be involved in adaptations to high altitudes and for bamboo consumption in other vertebrates. Notably, the mutations (Ala665Ser and Thr/Met667Val) in the *EPAS1* gene identified in both CF and FF is absent in other *Chlorocebus* species. *EPAS1* is a well-studied gene that codes for the hypoxia-inducible factor 2α (HIF-2α), which is involved in cellular responses to low oxygen (hypoxia) [27,31,90,91,92,93,94,95]. This shared mutation suggests a common adaptation for living at high altitudes in both CF and FF. Furthermore, the presence of mutations in *EPAS1* in other high-altitude mammals, such as Tibetans, Andeans, and Ethiopians [27,31,96,97], snub-nosed monkeys (*Rhinopithecus*) [29,34], and geladas (*Theropithecus gelada*) [33] indicates the important role of *EPAS1* in hypoxia adaptation.

Another intriguing finding is the unique mutation (Lys68Gln) in the *TAS2R16* gene found exclusively in the CF individual and the giant panda (*Ailuropoda melanoleuca*) [74,98,99,100]. *TAS2R16* encodes a bitter taste receptor that detects β-glucosides, which are abundant in cyanogenic plants like bamboo [101,102]. Previous research indicates that these taste receptors can influence food choices in pandas [103]. In giant and red pandas (*Ailurus fulgens*) and bamboo lemurs (*Hapalemur* and *Prolemur*), the deregulation of *TAS2R16* and other *TAS2R* taste receptor genes may reduce their sensitivity to bitterness. Such a reduced sensitivity may allow them to consume larger quantities of bamboo compared to other mammals [74,98,99,102,104,105]. The mutation in *TAS2R16* in the CF individual might alter the perception of bamboo’s bitter taste, potentially explaining a higher bamboo intake (80% of its diet) compared to FF and other *Chlorocebus* species [25].

It is likely that the bamboo-rich diet of CF results in a high degree of exposure to cyanogenic compounds [35]. The *TST* and *MPST* genes are crucial for cyanide detoxification [81,106]. We found more unique non-synonymous mutations in these genes in the CF individual (seven amino acid changes) compared to the FF individual (two amino acid changes). The high cyanide content in bamboo underscores the importance of efficient detoxification pathways for survival in a bamboo-rich environment. Therefore, the larger number of mutations in *TST* and *MPST* genes in the CF individual may represent genetic adaptations that enhance cyanide detoxification, supporting their ability to survive on a predominantly bamboo-rich diet.

## 5. Conclusions

We revealed *C. djamdjamensis* as a sister species to *C. aethiops* and confirmed gene flow between *C. aethiops* and the investigated FF individual but not with the CF individual, supporting previous gene flow hypotheses based on mitochondrial DNA and phenotypic information. We identified several non-synonymous mutations unique to *C. djamdjamensis* in genes related to high altitudes and bamboo consumption. However, we found fewer of these mutations in the FF compared to the CF individual. The potential loss of such private alleles in FF might be due to introgression and nuclear swamping by congeners. The functional consequences of these mutations remain unknown and need further investigation, which may offer insights into the adaptation mechanisms of *C. djamdjamensis* to their ecological niche. Our results are based on only one individual from each site and consequently should be regarded as preliminary. In the future, a larger number of *C. djamdjamensis* samples with a higher sequencing depth are needed to verify and refine our findings.

## Data Availability

Short-read sequencing data are available through NCBI repositories and are linked to BioProject accession number PRJNA1137408.

## Supplementary Materials

The following supporting information can be downloaded at: https://www.mdpi.com/article/10.3390/genes15111359/s1. Table S1. Sample information and mapping statistics (to Mmul_10) of the 42 investigated *Chlorocebus* individuals. Table S2. Autosome-wide heterozygosity for *Chlorocebus* species, measured as number of heterozygous sites per 1000 bp. Table S3. Results of ABBA-BABA test with Dsuite (version 0.5 r57) software, testing for gene flow among all *Chlorocebus* taxa, with *C. sabaeus* as outgroup. Table S4. Results of chromosome-based, 3-taxon test performed with *D_FOIL_*, for the trio CF, FF, and *C. aethiops* (*p* < 0.05). Table S5. Results of QuIBL analysis with three populations and probability of presence of only ILS (mixprop1) or ILS and introgression (mixprop2). Table S6. Number and percent of SNPs per variant type for the two *C. djamdjamensis* individuals mapped to *C. sabaeus* (chlsab2) reference genome. Table S7. Candidate genes related to high-altitude adaptation and bamboo metabolism as revealed by earlier studies. Table S8. Gene Ontology (GO) terms related to high-altitude adaptation and bamboo metabolism as obtained by enrichment analysis (corrected *p* < 0.05). Table S9. KOBAS gene enrichment for FF *C. djamdjamensis* (corrected *p* < 0.05). Table S10. KOBAS gene enrichment for CF *C. djamdjamensis* (corrected *p* < 0.05). Figure S1. Mitochondrial phylogeny of *Chlorocebus* based on complete mitochondrial genomes. Figure S2. Three-dimensional PCA of *Chlorocebus* species. The variance explained by PC 1 is 10.5%, 9.7% by PC2, and 7.3% by PC3. Figure S3. PSMC plots for investigated *Chlorocebus* individuals. Figure S4. DensiTree visualization of autosomal window trees (500kb window size and 250 kb steps). Figure S5. Frequency of observed branching patterns between *C. aethiops* and *C. djamdjamensis* based on 50kb window trees. Figure S6. Partial amino acid alignments of the thiosulfate sulfurtransferase (TST) showing amino acid changes in either CF and FF or only in the CF *C. djamdjamensis* individual. Figure S7. Partial amino acid alignments of the mercaptopyruvate sulfurtransferase (MPST) with amino acid changes in only the CF *C. djamdjamensis* individual. Refs. [107,108,109,110,111,112,113,114,115] are cited in Supplementary Materials.
