# Supplementary material for: Whole Genome Analysis Reveals Evolutionary History and Introgression Events in Bale Monkeys
Source: Genes (Basel). 2024 Oct 23;15(11):1359. doi: 10.3390/genes15111359 (PMC11593718; doi:10.3390/genes15111359)
Supplement: Supplementary file 1 [file genes-15-01359-s001.zip › SupplMaterials_Oct22.docx]

**Supplementary Material**

**Whole Genome Analysis Reveals Evolutionary History and Introgression Events in Bale Monkeys**

Lakshmi Seshadri^1,2^, Anagaw Atickem^3^, Dietmar Zinner^4,5,6^, Christian Roos^1,7,*^, Liye Zhang^1,*^

^1^ Primate Genetics Laboratory, German Primate Center, Leibniz Institute for Primate Research, 37077 Göttingen, Germany; [lseshadri@dpz.eu](mailto:lseshadri@dpz.eu)

^2^ International Max Planck Research School for Genome Science (IMPRS-GS), Georg-August-Universität Göttingen, 37077 Göttingen, Germany

^3^ Department of Zoological Sciences, Addis Ababa University, Addis Ababa 999047, Ethiopia; [anagawam@gmail.com](mailto:anagawam@gmail.com)

^4^ Cognitive Ethology Laboratory, German Primate Center, Leibniz Institute for Primate Research, 37077 Göttingen, Germany; [dzinner@gwdg.de](mailto:dzinner@gwdg.de)

^5^ Department of Primate Cognition, Georg-August-Universität Göttingen, 37077 Göttingen, Germany

^6^ Leibniz ScienceCampus Primate Cognition, 37077 Göttingen, Germany

^7^ Gene Bank of Primates, German Primate Center, Leibniz Institute for Primate Research, 37077 Göttingen, Germany

* Correspondance: [lzhang@dpz.eu](mailto:lzhang@dpz.eu), [croos@dpz.eu](mailto:croos@dpz.eu)

**Table S1.** Sample information and mapping statistics (to Mmul_10) of the 42 investigated *Chlorocebus* individuals. (This table can be found in Supplementary Excel file)

**Table S2.** Autosome-wide heterozygosity for *Chlorocebus* species, measured as number of heterozygous sites per 1000 bp.

| Species | Heterozygosity | Mean |
| --- | --- | --- |
| *C. djamdjamensis* FF | 1.11 | - |
| *C. djamdjamensis* CF | 0.68 | - |
| *C. aethiops* | 0.52 – 1.12 | 0.78 |
| *C. p. hilgerti* | 0.79 – 0.86 | 0.83 |
| *C. p. pygerythrus* | 0.65 – 0.95 | 0.76 |
| *C. cynosuros* | 0.57 – 0.90 | 0.81 |
| *C. tantalus* | 0.77 – 0.96 | 0.87 |
| *C. sabaeus* | 0.75 – 0.85 | 0.80 |

**
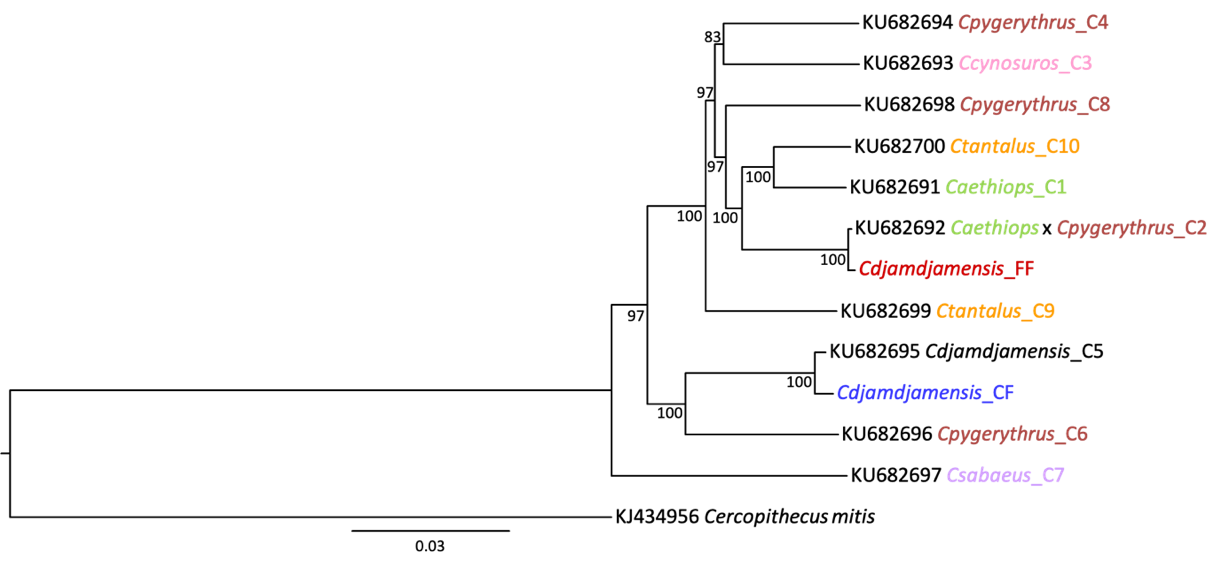
**

**Figure S1.** Mitochondrial phylogeny of *Chlorocebus* based on complete mitochondrial genomes. Given are GenBank accession number, species, and clade designation following Haus et al. [21] and Dolotovskaya et al. [42]. Numbers at nodes refer to bootstrap values and the bar below the tree indicates substitutions per site. The topology and the placement of the two Bale monkeys in haplogroups C5 and C2 are congruent with mitochondrial phylogenies of previous studies [21,23,42].


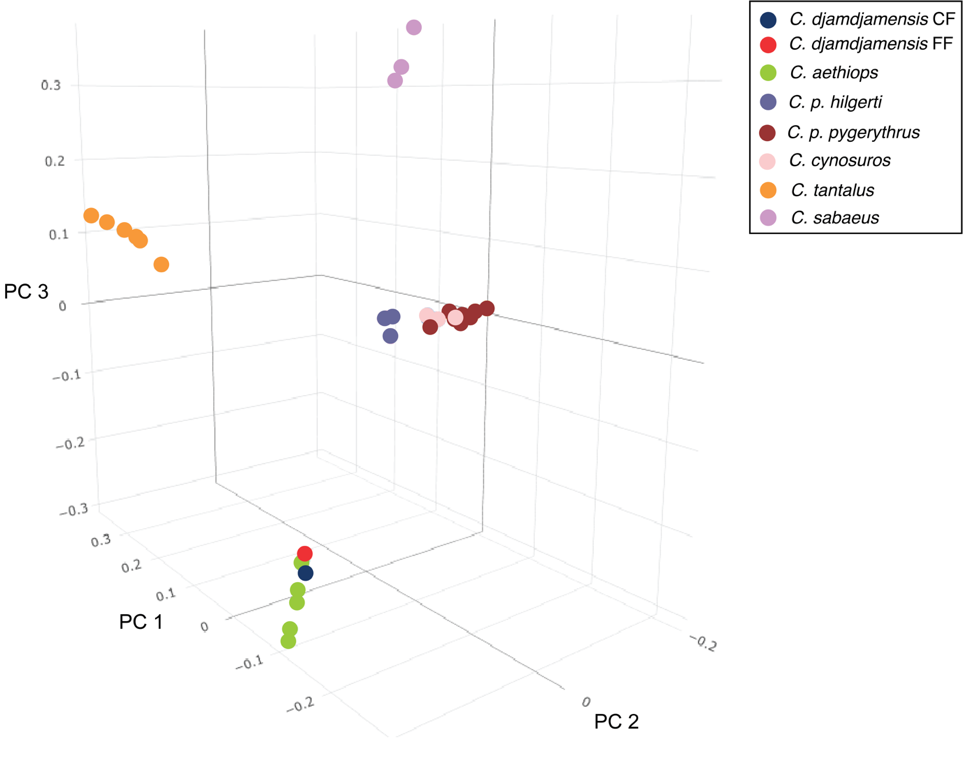


**Figure S2.** Three-dimensional PCA of *Chlorocebus* species. The variance explained by PC 1 is 10.5%, 9.7% by PC2, and 7.3% by PC3.


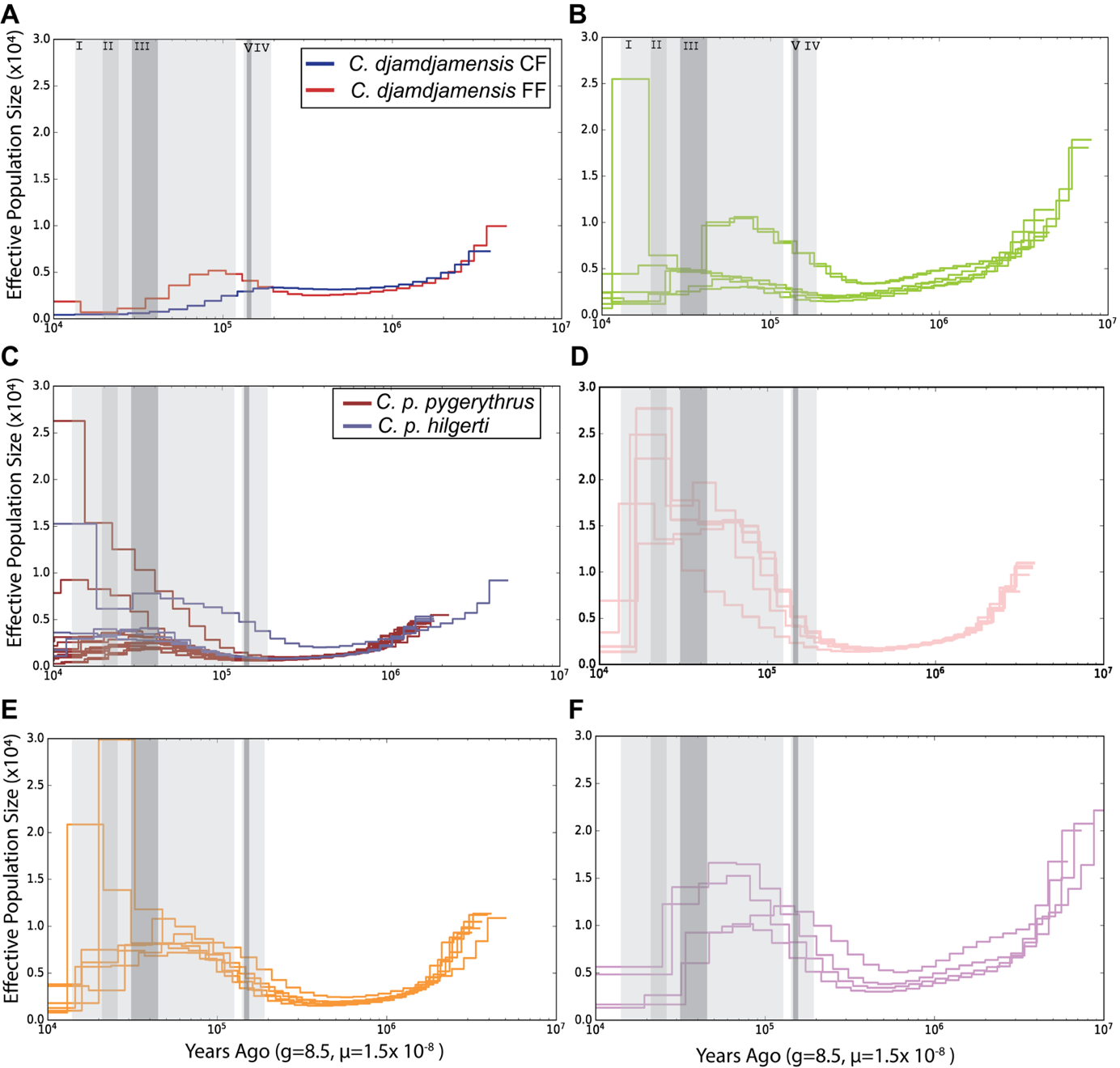


**Figure S3.** PSMC plots for investigated *Chlorocebus* individuals. Grey shaded areas are (I) last glacial period, (II) last glacial maximum, (III) last glacial maximum in the Bale mountains, (IV) penultimate glacial period, and (V) penultimate glacial maximum. **(A)** *C. djamdjamensis* CF and FF, **(B)** *C. aethiops*, **(C)** *C. p. pygerythrus* and *C.p. hilgerti,* **(D)** *C. cynosuros*, **(E)** *C. tantalus* and **(F)** *C. sabaeus*. The reconstructions are based on a mutation rate of 1.5 × 10^-8^ and a generation time of 8.5 years.

**Table S3.** Results of ABBA-BABA test with Dsuite software (version 0.5 r57), testing for gene flow among all *Chlrorocebus* taxa, with *C. sabaeus* as outgroup. Gene flow between FF and *C. aethiops* is marked in yellow.

| **P1** | **P2** | **P3** | **D-statistic** | **Z-score** | **p-value** | **f4-ratio** |
| --- | --- | --- | --- | --- | --- | --- |
| CF | FF | *C. aethiops* | 0.0951086 | 15.3347 | 2.30E-16 | 0.146772 |
| FF | CF | *C. cynosuros* | 0.0374243 | 14.5485 | 2.30E-16 | 0.0289071 |
| FF | CF | *C. p. hilgerti* | 0.0266853 | 8.16738 | 3.15E-16 | 0.0193068 |
| FF | CF | *C. p. pygerythrus* | 0.0347816 | 13.2151 | 2.30E-16 | 0.0321307 |
| FF | CF | *C. tantalus* | 0.0189849 | 9.57851 | 2.30E-16 | 0.0164621 |
| CF | *C. aethiops* | *C. cynosuros* | 0.0156684 | 6.49067 | 8.55E-11 | 0.0188317 |
| *C. aethiops* | CF | *C. p. hilgerti* | 0.00969277 | 3.40921 | 0.00065151 | 0.0105477 |
| CF | *C. aethiops* | *C. p. pygerythrus* | 0.0269944 | 10.3498 | 2.30E-16 | 0.039115 |
| CF | *C. aethiops* | *C. tantalus* | 0.0406326 | 25.6004 | 2.30E-16 | 0.0549706 |
| *C. cynosuros* | *C. p. hilgerti* | CF | 0.0293994 | 21.4679 | 2.30E-16 | 0.014312 |
| *C. p. pygerythrus* | *C. cynosuros* | CF | 0.00514362 | 5.81089 | 6.21E-09 | 0.00239934 |
| CF | *C. cynosuros* | *C. tantalus* | 0.0701316 | 38.1508 | 2.30E-16 | 0.130296 |
| *C. p. pygerythrus* | *C. p. hilgerti* | CF | 0.0341919 | 28.7529 | 2.30E-16 | 0.016677 |
| CF | *C. p. hilgerti* | *C. tantalus* | 0.0773884 | 44.0111 | 2.30E-16 | 0.140044 |
| CF | *C. p. pygerythrus* | *C. tantalus* | 0.0725302 | 38.3233 | 2.30E-16 | 0.136014 |
| FF | *C. aethiops* | *C. cynosuros* | 0.0449289 | 18.1858 | 2.30E-16 | 0.0471924 |
| FF | *C. aethiops* | *C. p. hilgerti* | 0.0090927 | 3.46597 | 0.00052832 | 0.00885198 |
| FF | *C. aethiops* | *C. p. pygerythrus* | 0.0553629 | 20.6607 | 2.30E-16 | 0.0699796 |
| FF | *C. aethiops* | *C. tantalus* | 0.0586538 | 32.9968 | 2.30E-16 | 0.0705255 |
| *C. cynosuros* | *C. p. hilgerti* | FF | 0.0374757 | 28.4069 | 2.30E-16 | 0.0218939 |
| *C. p. pygerythrus* | *C. cynosuros* | FF | 0.00378245 | 2.9621 | 0.00305547 | 0.00211065 |
| *C. tantalus* | *C. cynosuros* | FF | 0.0716865 | 29.1124 | 2.30E-16 | 0.0461138 |
| *C. p. pygerythrus* | *C. p. hilgerti* | FF | 0.040864 | 28.4645 | 2.30E-16 | 0.0239583 |
| FF | *C. p. hilgerti* | *C. tantalus* | 0.0897429 | 55.1278 | 2.30E-16 | 0.154196 |
| *C. tantalus* | *C. p. pygerythrus* | FF | 0.0683307 | 30.2308 | 2.30E-16 | 0.0440962 |
| *C. cynosuros* | *C. p. hilgerti* | *C. aethiops* | 0.00705954 | 7.46036 | 8.63E-14 | 0.00768121 |
| *C. cynosuros* | *C. p. pygerythrus* | *C. aethiops* | 0.00484157 | 6.52973 | 6.59E-11 | 0.00512983 |
| *C. aethiops* | *C. cynosuros* | *C. tantalus* | 0.038965 | 24.7204 | 2.30E-16 | 0.0797213 |
| *C. p. pygerythrus* | *C. p. hilgerti* | *C. aethiops* | 0.00232866 | 2.72221 | 0.00648464 | 0.00256459 |
| *C. aethiops* | *C. p. hilgerti* | *C. tantalus* | 0.0446047 | 29.3519 | 2.30E-16 | 0.0900381 |
| *C. aethiops* | *C. p. pygerythrus* | *C. tantalus* | 0.0417967 | 26.046 | 2.30E-16 | 0.0857724 |
| *C. p. pygerythrus* | *C. cynosuros* | *C. p. hilgerti* | 0.00174928 | 2.52631 | 0.0115267 | 0.0035015 |
| *C. cynosuros* | *C. p. hilgerti* | *C. tantalus* | 0.00557028 | 8.2008 | 2.39E-16 | 0.0112084 |
| *C. cynosuros* | *C. p. pygerythrus* | *C. tantalus* | 0.0033428 | 5.28115 | 1.28E-07 | 0.00657398 |
| *C. p. pygerythrus* | *C. p. hilgerti* | *C. tantalus* | 0.00228504 | 2.99126 | 0.00277826 | 0.0046666 |

**Table S4.** Results of chromosome-based, 3-taxon test performed with DFOIL, for the trio CF, FF, and *C. aethiops* (*p* < 0.05).

| **chrom** | **D_left** | **D_right** | **D_stat** |
| --- | --- | --- | --- |
| chr1 | 13906 | 9950 | 0.1658283 |
| chr10 | 6966 | 4178 | 0.25017947 |
| chr11 | 8764 | 5787 | 0.20459075 |
| chr12 | 10881 | 7923 | 0.15730696 |
| chr13 | 8379 | 5709 | 0.189523 |
| chr14 | 8394 | 5934 | 0.17169179 |
| chr15 | 7287 | 5513 | 0.13859375 |
| chr16 | 3527 | 2589 | 0.15336821 |
| chr17 | 10626 | 6717 | 0.22539353 |
| chr18 | 8089 | 5131 | 0.22375189 |
| chr19 | 2131 | 1457 | 0.18784838 |
| chr2 | 14934 | 11589 | 0.12611696 |
| chr20 | 5977 | 3715 | 0.23338836 |
| chr3 | 16252 | 12107 | 0.14616171 |
| chr4 | 15469 | 11396 | 0.1516099 |
| chr5 | 17247 | 12960 | 0.14192075 |
| chr6 | 16134 | 12115 | 0.14227052 |
| chr7 | 11451 | 8551 | 0.1449855 |
| chr8 | 12380 | 9065 | 0.15458149 |
| chr9 | 12458 | 8462 | 0.19101338 |
|  |  |  |  |


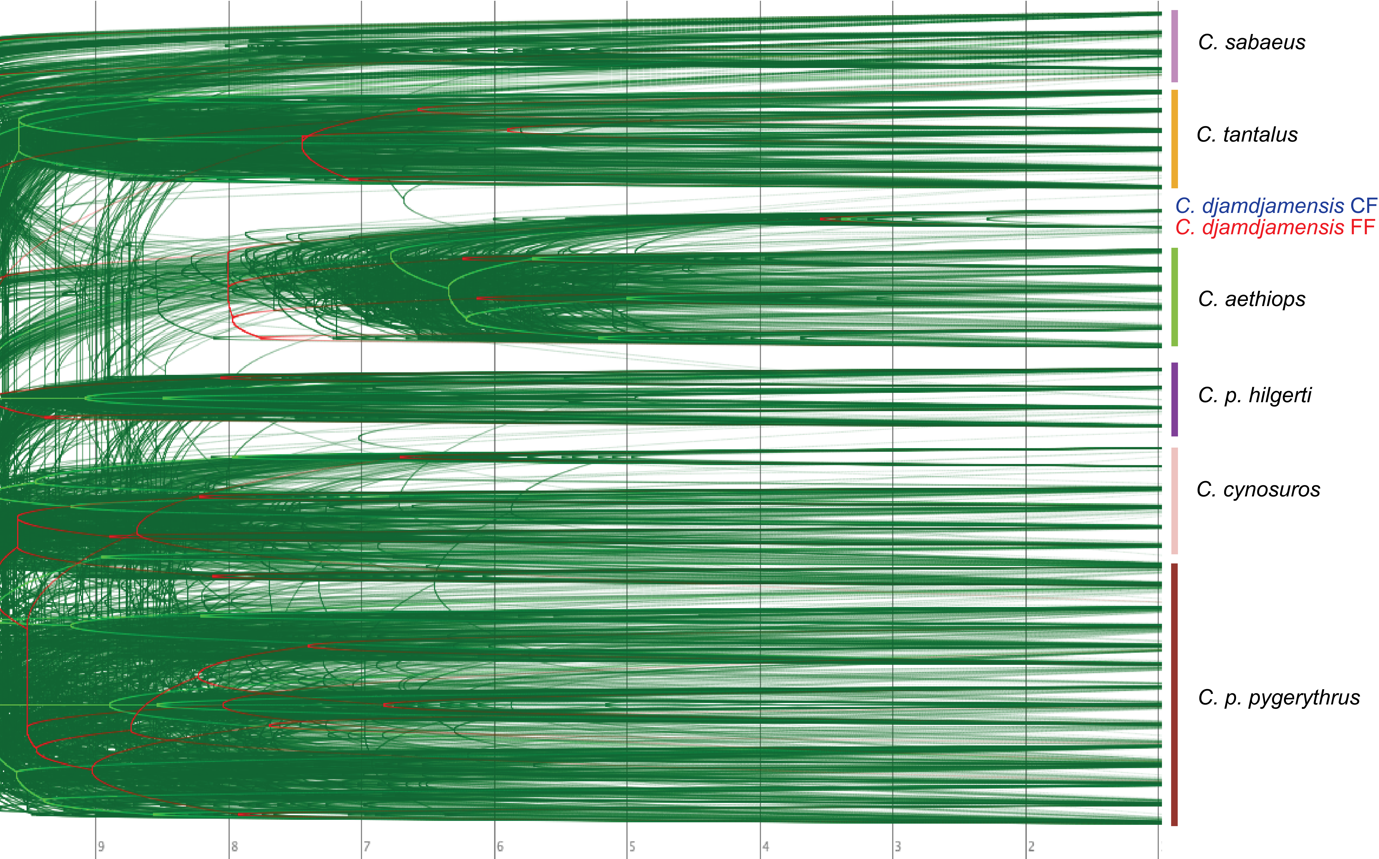


**Figure S4.** DensiTree visualization of autosomal window trees (500kb window size and 250 kb steps).


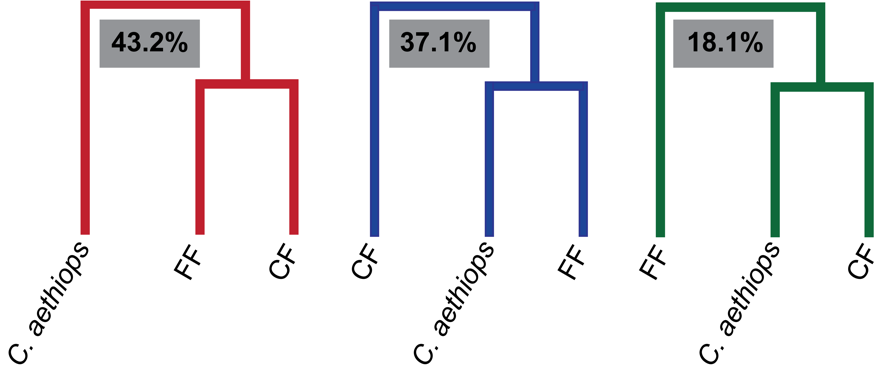


**Figure S5.** Frequency of observed branching patterns between *C. aethiops* and *C. djamdjamensis* based on 50kb window trees.

**Table S5.** Results of QuIBL analysis with three populations and probability of presence of only ILS (mixprop1) or ILS and introgression (mixprop2). Combinations of *C. aethiops* with FF are marked in yellow. Additionally, combination of FF with geographically closest *C. aethiops* samples, aethiops_04 and aethiops_05, are marked in orange. (This table can be found in Supplementary Excel file)

**Table S6.** Number and percent of SNPs per variant type for the two *C. djamdjamensis* individuals mapped to *C. sabaeus* (chlsab2) reference genome.

| **Mutation Effect** | *C. djamdjamensis* FF | | *C. djamdjamensis* CF | |
| --- | --- | --- | --- | --- |
|  | Counts | % | Counts | % |
| 3’ UTR variant | 78,766 | 0.38 | 75,033 | 0.38 |
| 5’ UTR start codon gain variant | 1,774 | 0.01 | 1,646 | 0.01 |
| 5’ UTR variant | 12,589 | 0.06 | 11,861 | 0.06 |
| Downstream gene variant | 828,576 | 4.04 | 76,3487 | 3.92 |
| Frameshift variant | 492 | 0.002 | 465 | 0.002 |
| Initiator codon variant | 5 | 0 | 4 | 0 |
| Intergenic region | 13,386,246 | 65.27 | 12,851,922 | 65.95 |
| Intron variant | 5,103,789 | 24.89 | 4,606,354 | 23.64 |
| Nonsynonymous variant | 39,141 | 0.19 | 39,008 | 0.20 |
| Noncoding transcript exon variant | 8,550 | 0.04 | 8,169 | 0.04 |
| Noncoding transcript variant | 28 | 0 | 25 | 0 |
| Splice acceptor variant | 270 | 0.001 | 287 | 0.001 |
| Splice donor variant | 211 | 0.001 | 199 | 0.001 |
| Splice region variant | 10,339 | 0.05 | 10,387 | 0.05 |
| Start lost | 55 | 0 | 47 | 0 |
| Stop gained | 376 | 0.002 | 403 | 0.002 |
| Stop lost | 69 | 0 | 67 | 0 |
| Stop retained variant | 33 | 0 | 33 | 0 |
| Synonymous variant | 52,471 | 0.26 | 52,814 | 0.27 |
| Upstream gene variant | 985,211 | 4.80 | 1,065,730 | 5.47 |

**Table** **S7.** Candidate genes related to high-altitude adaptation and bamboo metabolism as revealed by earlier studies.

| **High-Altitude Adaptation** | **Bamboo Metabolism** |
| --- | --- |
| *ACE* [107] | *BGLU* [101] |
| *ADH1B* [108] | *CPT1* [114] |
| *ADAM9* [30] | *CYP2A* [114] |
| *CXCLIO* [109] | *CYP2B* [114] |
| *ENGLN1* [27] | *MPST* [105] |
| *EPAS1* [27,90,92,93] | *TASR16* [74,99,102] |
| *EPOR* [110] | *TASR24* [74,99] |
| *ERBIN* [109] | *TASR42* [74,99] |
| *ESYT3* [91] | *TH* [74] |
| *FYCOI* [109] | *T1R1* [103] |
| *HBB* [95] | *TST* [105] |
| *HIF1A* [90] | *UGT* [115] |
| *HSPA1A* [111] |  |
| *NOS3* [95] |  |
| *PPARA* [27,93] |  |
| *PRKAA1* [97] |  |
| *RORA* [29] |  |
| *SLC2A1* [112] |  |
| *VEGFA* [112] |  |
| *VAV3* [113] |  |

**Table** **S8.** Gene Ontology (GO) terms related to high-altitude adaptation and bamboo metabolism as obtained by enrichment analysis (corrected *p* < 0.05). Highlighted in yellow are the terms described in the main text.

| **High-Altitude Adaptation** | **Bamboo Metabolism** |
| --- | --- |
| Response to hypoxia | Toxin transport |
| HIF activation | Retinol metabolism |
| Vasodilation (only FF) | Retinol metabolic process |
| Angiogenesis | Xenibiotic detoxification (only FF) |
| Regulation of angiogenesis |  |
| Cellular response to oxidative stress |  |
| Cellular response to hypoxia |  |

**Table** **S9.** KOBAS gene enrichment for FF *C. djamdjamensis* (corrected *p* < 0.05). Yellow markings highlight important pathaways and GO terms related to high-altitude living and bamboo metabolism. (This table can be found in Supplementary Excel file).

**Table** **S10.** KOBAS gene enrichment for CF *C. djamdjamensis* (corrected *p* < 0.05). Yellow markings highlight important pathways and GO terms related to high-altitude living and bamboo metabolism. (This table can be found in Supplementary Excel file).


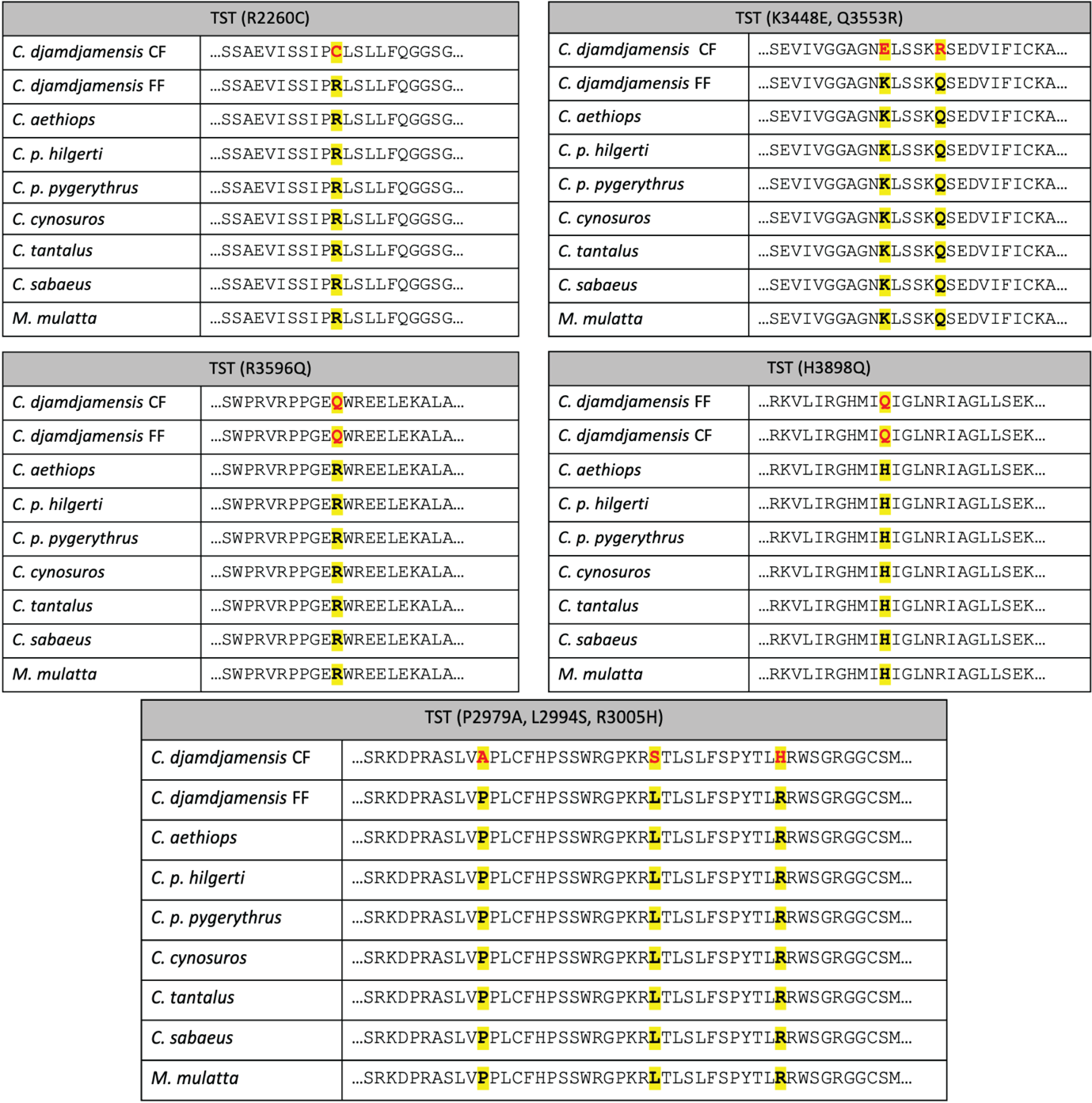


**Figure S6.** Partial amino acid alignments of the thiosulfate sulfurtransferase (*TST*) showing amino acid changes in either CF and FF or only in the CF *C. djamdjamensis* individual. Yellow markings indicate the non-synonymous mutations that differentiate *C. djamdjamensis* from other *Chlorocebus* species and *M. mulatta*.

**
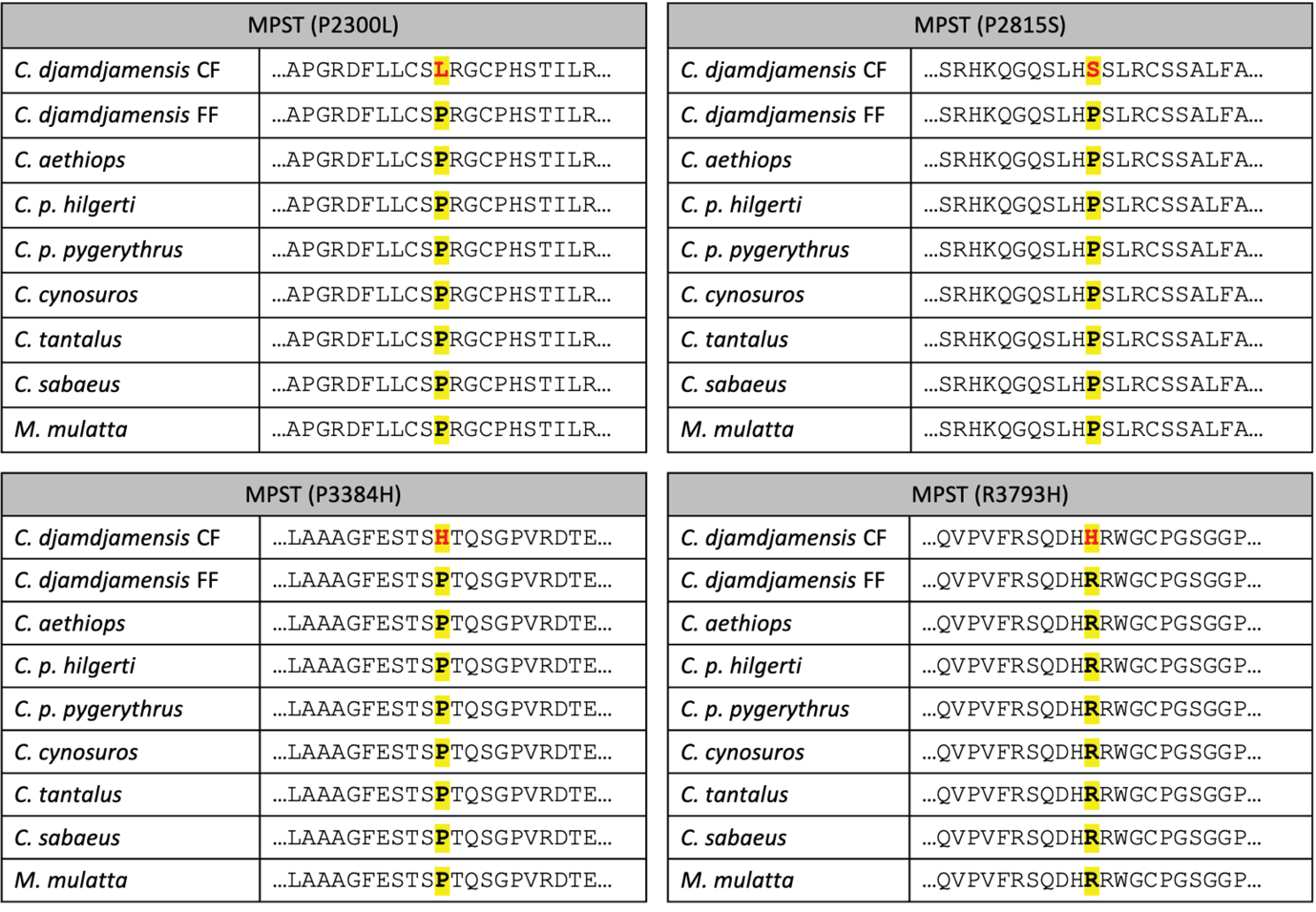
**

**Figure S7.** Partial amino acid alignments of the mercaptopyruvate sulfurtransferase (*MPST*) with amino acid changes in only the CF *C. djamdjamensis* individual. Yellow markings indicate the non-synonymous mutations that differentiate CF *C. djamdjamensis* from other *Chlorocebus* species and *M. mulatta*.
